# Treatment-independent miRNA signature in blood of wilms tumor patients

**DOI:** 10.1186/1471-2164-13-379

**Published:** 2012-08-07

**Authors:** Jana Schmitt, Christina Backes, Nasenien Nourkami-Tutdibi, Petra Leidinger, Stephanie Deutscher, Markus Beier, Manfred Gessler, Norbert Graf, Hans-Peter Lenhof, Andreas Keller, Eckart Meese

**Affiliations:** 1 Department of Human Genetics, Saarland University, 66421 Homburg/Saar, Germany; 2 Department of Pediatric Oncology and Hematology, Medical School, Saarland University, 66421 Homburg, Germany; 3 febit group, 69120 Heidelberg, Germany; 4 Developmental Biochemistry, Biocenter, University of Würzburg, 97074 Würzburg, Germany; 5 Center for Bioinformatics, Saarland University, 66041 Saarbrücken, Germany

## Abstract

**Background:**

Blood-born miRNA signatures have recently been reported for various tumor diseases. Here, we compared the miRNA signature in Wilms tumor patients prior and after preoperative chemotherapy according to SIOP protocol 2001.

**Results:**

We did not find a significant difference between miRNA signature of both groups. However both, Wilms tumor patients prior and after chemotherapy showed a miRNA signature different from healthy controls. The signature of Wilms tumor patients prior to chemotherapy showed an accuracy of 97.5% and of patients after chemotherapy an accuracy of 97.0%, each as compared to healthy controls.

**Conclusion:**

Our results provide evidence for a blood-born Wilms tumor miRNA signature largely independent of four weeks preoperative chemotherapy treatment.

## Background

Wilms tumor is a common childhood tumor and affects very young children [1]. Most of cases are detected at the median age of 36 months. In Europe, children older than six months are treated with preoperative chemotherapy according to the SIOP protocol (Societé Internationale d’Oncologie Pédiatrique) resulting in a smaller tumor volume when children undergo surgery. The primary aims of the SIOP study are risk-stratified therapies, dimished toxicity and an improved patient outcome. Since children in early stages of Wilms tumor development do not show characteristic symptoms, and low grade tumors have a better prognosis than high grade tumors [2], an early detection marker is useful to increase the overall survival rates. Most recently, we provided strong evidence that blood-born microRNA (miRNA) signatures are highly indicative for various human diseases [3]. MiRNAs are small non-coding RNAs between 17 and 24 nt. They are involved in physiological and pathological processes through the regulation of gene expression and may indicate tumor development at very early stages [4,5]. Specific miRNA signatures have already been described for clear cell renal cell carcinoma (ccRCC) [6]. They allow to differentiate metastatic and non-metastatic ccRCC and indicate progression-free survival and overall survival. MiRNA profiles of different human kidney cancer subtypes have been established by Petillo et al. [7]. They were able to discriminate chromophobe RCC from oncocytomas, ccRCC from papillary RCC and prognostic subgroups of ccRCC. While these renal miRNA patterns have been established on tumor tissues, in our study we analyzed the miRNA expression pattern in blood cells of Wilms tumor patients. Specifically we compared untreated children with Wilms tumor and children treated according to SIOP protocol. We asked if chemotherapy affects the miRNA expression patterns. In addition, we asked if these expression patterns are different between Wilms tumor patients and healthy controls.

## Methods

### Samples

In total, we collected more than hundred blood samples from Wilms tumor patients. To arrvive at homogenous patient groups, we excluded samples taken from patients after primary surgery, from patients with relapses, from patients after post-chemotherapy surgery and from patients with a non-Wilms tumor histology as determined by biopsy. We obtained 43 Wilms tumor samples for analysis, including 23 samples taken from Wilms tumor patients prior to chemotherapy and 20 samples from patients after chemotherapy. Out of these were 26 matched samples, i.e. 13 samples prior to chemotherapy and 13 samples after chemotherapy of the same patients. The mean age of the treated patients was 3.3 years +/- 2.2, the mean age of the untreated patients was 4.9 years +/- 3.9. The collection contained 41 samples of unilateral and 2 samples of bilateral tumors. Detailed patient information is available in Additional file 1: Table S1. We also collected blood samples with the consent of 19 healthy controls. The mean age of healthy controls was 37.8 years +/- 14.2. In each case, 2.5 ml peripheral blood was collected in PAXgene Blood RNA tubes (BD, Franklin Lakes, NJ, USA) and stored at -20°C. Blood samples were obtained with parents’ informed consent from the Department of Pediatric Hematology and Oncology of the Saarland University and from the multicenter study SIOP 2001/GPOH. The study was approved by the local ethics committee (No.136/01). Detailed information is available in Additional file 1: Table S2.

### RNA isolation and microarray screening

To isolate the RNA including the miRNA from whole blood samples, we used the miRNeasy Kit (Qiagen GmbH, Hilden) as previously described [8]. We analyzed the total RNA by using the Geniom RT Analyzer (febit biomed GmbH, Heidelberg, Germany) employing the MPEA-assay for miRNA-analysis. This assay allows for detection of microRNAs based on a combination of stringent hybridization and enzymatic primer extension on a microfluidic microarray starting from total RNA material, without the need for enrichment, amplification or labeling of the native RNA samples [9]. The microfluidic biochip (Geniom Biochip Homo sapiens v12, febit biomed GmbH, Heidelberg, Germany) contained 7 replicates of 848 miRNAs [10,11] as annotated in the Sanger miRBase version 12.0 (http://www.mirbase.org). The expression profiles are deposited in the GEO (Gene Expression Omnibus) database under the accession number GSE38419.

### Quantitative Real-Time PCR

Relative quantification Real-Time PCR (qRT-PCR) was performed to confirm the array results on an a StepOnePlus^™^ Real-Time PCR System (Applied Biosystems, Foster City, USA). The miScript PCR System (Qiagen, Valencia, CA, USA) was used and all procedures were carried out according to manufacturer’s recommendations. Briefly, 250 ng of total RNA containing miRNAs was mixed with 4 *μl* of miScript RT Buffer and 1 *μl*miScript Reverse Transcriptase mix, RNase-free water in a total volume of 20 *μl* (Qiagen, Valencia, CA, USA). Samples were further incubated at 37°C for 60 min for the first strand cDNA synthesis. Thereafter, the reaction was inactivated by heating at 95°C for 15 min and the resulting cDNA was stored at -20°C until analysis. The cDNA converted from total RNA containing miRNAs served as the template for Real-time PCR analysis using the miScript SYBR Green PCR Kit along with the 10x miScript Primer Assays for hsa-miR-520d-3p, hsa-miR-197, hsa-miR-224, hsa-miR-20a, hsa-miR-126, and hsa-miR-144* (Qiagen, Valencia, CA, USA). Each PCR reaction contained: 2.5 *μl* cDNA, 12.5 *μl* 2x QuantiTect SYBR Green PCR Master Mix, 2.5 *μl* 10x miScript Universal Primer, 2.5 *μl*10x miScript Primer Assay and RNase-free water to a total volume of 25 *μl*, and placed into the individual wells of a 96-well plate. Reactions were run with the following thermal cycling parameters: initial activation step 95°C for 15 min followed by 40 cycles at 94°C for 15 sec (denaturation), 55°C for 30 sec (annealing) and 70°C for 30 sec (extension). Then a final dissociation curve (melting curve) was made, and PCR plates were kept at 4°C until they were taken out from the PCR machine. The RNU6B snRNAs primer assay (Qiagen, Valencia, CA, USA) was chosen as an endogenous reference for normalization studies.

### Statistical analysis

The background corrected and log transformed microarrays were quantile normalized using the freely available R software [12]. Differentially expressed miRNAs were identified by employing an unpaired two-tailed t-test. The resulting p-values were adjusted for multiple testing by applying the Benjamini-Hochberg procedure (false discovery rate (FDR) adjustment) [13]. In addition, we computed AUC (Area Under the Curve) values for each miRNA. AUC values measure how well the expression values of a single miRNA can separate two groups, e.g. patients versus controls. An AUC value of 0.5 indicates equal distribution of the two groups to compare; this means that a miRNA cannot be used to separate the groups. A miRNA reaching an AUC value of 1 (0) has expression values that are throughout lower (higher) in the diseased group than in the control group corresponding to the most diagnostic information of a biomarker. Besides this single biomarker analysis, we classified the samples using the normalized and log transformed miRNA expression profile with machine learning procedures for calculating Support Vector Machines (SVM) implemented in the R e1071 package. We performed the classification with a linear SVM employing 100 repetitions of standard 10-fold cross validation and calculated mean sensitivity, specificity, and accuracy. Clustering has been carried out using complete linkage hierarchical clustering. The Euclidian distance measure was employed to compute the dissimilarity of miRNA rows and sample columns independently of each other. We used the heatmap.2 function of the gplots R package to compute and plot the results of the clustering. For validating the microarray results, we applied quantitative real-time PCR on six selected miRNAs and computed the fold change by using the 2^−*ΔΔCt*^ method [14].

## Results

In total, we collected 43 Wilms tumor samples for analysis. To generate miRNA profiles, we screened 848 mature miRNA transcripts. After applying background correction, log transformation, and normalization procedures, we tested if the miRNA profile changes after chemotherapy. To this end, we applied the paired two-tailed t-test for the matched samples of 13 Wilms tumor patients before and after chemotherapy. We considered only miRNAs with more than a two-fold expression difference between the two groups. Using the remanining 214 miRNAs, our analysis did not indicate a significant difference in miRNA profiles of Wilms tumor patients prior to chemotherapy and Wilms tumor patients after chemotherapy. Specifically, we did not find significantly deregulated miRNAs using a significance threshold of 0.05 after FDR adjustment. In addition, we repeated the comparison of Wilms patients before and after therapy using all 43 samples and applying unpaired two-tailed t-test, which confirmed the results of the matched samples (no significant miRNA using a significance threshold of 0.05 and FDR adjustment). The lack of a difference between post- and pre-treatment samples prompted us to ask whether Wilms tumors are characterized by a blood-born miRNA signature. To this end, we compared Wilms tumor patients prior chemotherapy with healthy controls. In detail, we compared 23 samples from Wilms tumor patients prior to chemotherapy with 19 healthy controls. After FDR p-value adjustment at a significance level of 0.05 using unpaired two-tailed t-test, we found 176 significantly deregulated miRNAs including 79 down-regulated and 97 up-regulated miRNAs in blood cells of Wilms tumor patients. The most significantly up-regulated miRNAs were hsa-miR-766, hsa-miR-1246, hsa-miR-197, and hsa-miR-224 with an increased median expression of 3.0-9.6 fold. The most significantly down-regulated miRNAs hsa-miR20a, hsa-miR-20b, hsa-miR-144* and hsa-miR-144 displayed median expression factors of 2.9-6.9 in Wilms tumor patients compared to control samples. The 176 deregulated miRNAs including their fold change, p-values, and AUC values are available in Additional file 2: Table S1. An overview of the 20 most significantly deregulated miRNAs can be found in Table 1.

**Table 1:** The 20 most significant miRNAs in Wilms tumor patients before therapy

**MiRNA**	**Log median**	**Log median**	**Log difference**	**Fold**	**Ttest**	**Ttest adj**	**AUC**
	**Wilms**	**Control**					
hsa-miR-20a	10.124	12.929	-2.805	-6.988	7.01E-012	5.94E-009	0.977
hsa-miR-20b	10.057	12.410	-2.353	-5.108	2.49E-011	1.05E-008	0.984
hsa-miR-766	10.079	8.494	1.585	3.000	2.63E-009	3.29E-007	0.033
hsa-miR-144*	7.994	9.524	-1.531	-2.889	2.40E-009	3.29E-007	0.953
hsa-miR-144	8.967	11.506	-2.540	-5.815	2.71E-009	3.29E-007	0.954
hsa-miR-106a	11.506	13.639	-2.132	-4.385	2.14E-009	3.29E-007	0.950
hsa-miR-1246	6.007	2.737	3.270	9.649	1.36E-009	3.29E-007	0.059
hsa-miR-197	11.205	8.888	2.317	4.984	4.33E-009	4.59E-007	0.055
hsa-miR-224	6.403	3.745	2.658	6.310	5.20E-009	4.90E-007	0.043
hsa-miR-18a	9.168	11.205	-2.037	-4.104	6.75E-009	5.72E-007	0.962
hsa-miR-93	10.830	12.766	-1.936	-3.825	8.50E-009	6.55E-007	0.935
hsa-miR-17	11.839	13.273	-1.434	-2.702	1.20E-008	8.48E-007	0.962
hsa-miR-18b	7.044	8.901	-1.856	-3.621	2.02E-008	1.32E-006	0.982
hsa-miR-126	8.320	11.425	-3.104	-8.601	3.38E-008	2.05E-006	0.944
hsa-miR-520d-3p	4.497	2.134	2.363	5.145	4.13E-008	2.33E-006	0.061
hsa-miR-1305	4.877	6.898	-2.021	-4.058	5.14E-008	2.60E-006	0.966
hsa-miR-373	4.819	1.976	2.843	7.173	5.22E-008	2.60E-006	0.064
hsa-miR-106b	13.004	14.104	-1.100	-2.143	6.24E-008	2.94E-006	0.938
hsa-miR-1204	5.360	3.048	2.312	4.967	1.21E-007	5.42E-006	0.078
hsa-miR-374a	6.473	8.483	-2.010	-4.029	1.39E-007	5.91E-006	0.934

The 20 most significant miRNAs when comparing Wilms patients before therapy to healthy controls. Down-regulated fold-change is indicated by negative values, up-regulated fold-change is indicated by positive values.

**Figure 1:**
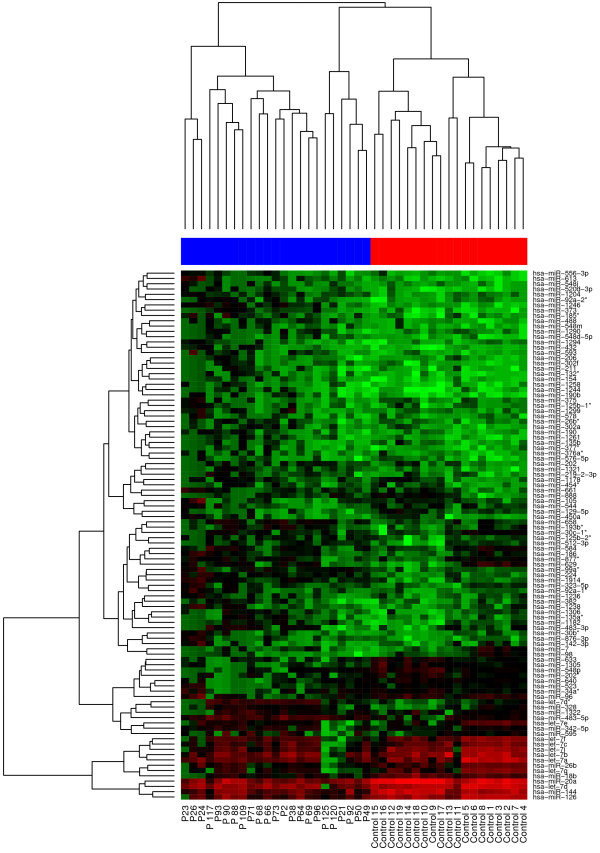
**Clustering of the 100 most variable miRNAs.** Clustering of the 100 most variable miRNAs for the classification between Wilms tumor patients prior to chemotherapy and healthy controls. Complete linkage hierarchical clustering has been performed with the Euclidian distance measure. Wilms patients and healthy controls cluster separately. The colors in the heatmap represent the normalized expression values with lower expression values being colored in shades of green and higher expression values in shades of red.

Using hierarchical clustering with the Euclidian distance measure, we analyzed how the 42 Wilms tumor and control samples relate to each other. For this task we used the 100 most variable miRNAs out of the 848 miRNAs. Figure 1 shows the resulting heatmap of the hierarchical clustering. The control samples clearly cluster different from the Wilms tumor samples. To determine if the expression pattern of the 848 miRNAs are able to separate Wilms tumor patients prior to chemotherapy from controls, we used a Support Vector Machine (SVM) with linear kernel. Briefly, we applied 100 repetitions of 10-fold cross-validations and computed the mean accuracy, sensitivity, specificity, and the corresponding 95% confidence intervals. Using this clustering, we were not able to separate Wilms tumor patients prior to chemotherapy and patients after chemotherapy, confirming the fold-expression results as shown above. But we were able to reach high values for the separation between blood samples taken from Wilms tumor patients prior to chemotherapy and samples taken from healthy controls with very low variances. In detail we found 97.5% accuracy (97.4 - 97.7%), 99.8% sensitivity (99.6 - 99.9%), and 94.7% specificity (94.6- 94.8%) for this separation.

**Figure 2:**
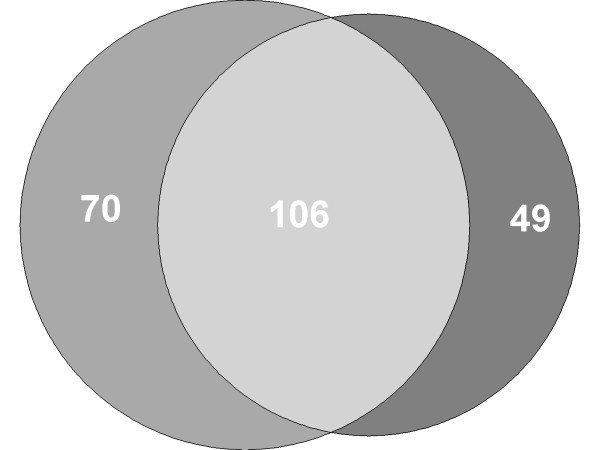
**Venn diagram of significantly deregulated miRNAs prior and after chemotherapy.** Venn diagram of miRNAs significantly deregulated in Wilms tumor patients prior and after chemotherapy compared to normal controls. There is an overlap of 106 miRNAs that are significantly deregulated in both groups. The overlap consists of 43 miRNAs that are in both comparisons down-regulated, and of 63 miRNAs that are in both comparisons upregulated.

Likewise we found comparable results for the separation between samples taken from Wilms tumor patients after chemotherapy and samples taken from controls. In detail we calculated an accuracy of 97.0% (96.7 - 97.3%) a sensitivity of 99.1% (98.5 - 99.7%) and a specificity of 94.8% (94.7 - 95.0%). These results support the abovementioned results that showed no significant difference of the blood-born miRNA signature between patients prior and after chemotherapy. Finally, we determined those miRNAs that are differently regulated in both comparisions between controls and samples taken prior to preoperative chemotherapy and between controls and samples taken after preoperative chemotherapy. We found a total of 106 miRNAs that were differentially regulated in both comparisons (adjusted t-test, significance level 0.05) (see Figure 2). Interestingly, the overlap consists of 43 miRNAs that are down-regulated and of 63 miRNAs that are up-regulated in both comparisons. These miRNAs are likely to contribute most to the treatment independent Wilms tumor signature.

**Figure 3:**
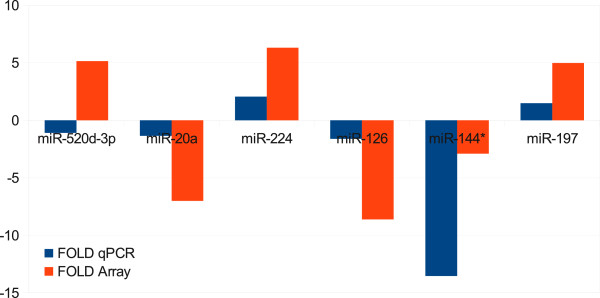
**Comparison of the fold changes of microarray experiment and qRT-PCR.** Comparison of the fold changes of six miRNAs found deregulated in Wilms patients in the microarray experiment and the corresponding qRT-PCR results.

To validate our microarry results, we selected six miRNAs for performing quantitative reverse transcription PCR (qRT-PCR). In total, we chose three miRNAs that were up-regulated (miR-520d-3p, miR-197, miR-224), and three that were down-regulated (miR-20a, miR-126, miR-144*) in the microarray experiment comparing Wilms tumor samples against normal controls. We performed the qRT-PCR in duplicates with 10 samples per group. The qRT-PCR results for the tested miRNAs were largely concordant with the microarray data (Figure 3), except for miR-520d-3p, which is down-regulated in qRT-PCR instead of its up-regulation in the microarry experiment.

## Discussion

As shown in several studies, miRNA expression signatures allow differentiation between human cancers [15,16]. There is also evidence that miRNA signatures correlate with metastasis, progression-free survival and overall survival [6]. As for human kidney cancer, specific signatures have been reported for clear cell renal cell carcinoma (ccRCC), papillary RCC and prognostic subgroups of ccRCC [7]. While the majority of studies focused on miRNAs in tissues, there is increasing evidence that miRNAs isolated from body fluids like serum, plasma or blood cells, can serve as an informative marker for different cancer types like ovarian cancer, prostate cancer, lung cancer, colorectal cancer as reviewed by Cortez et al. [17]. Most recently we reported specific miRNA signatures of peripheral blood cells in a variety of cancer and non-cancer diseases. In detail, we detected consistently deregulated profiles for lung cancer, prostate cancer, melanoma, ovarian cancer, gastric tumors, pancreatic tumors, multiple sclerosis, chronic obstructive pulmonary disease (COPD), sarcoidosis, periodontitis, pancreatitis and acute myocardial infarction [3]. Based on our data and the data of others [18], the overall pattern of miRNAs from blood cells seems to be rather robust. In addition, blood cells allow isolation of rather large amounts of miRNAs that in turn are sufficient for next generation sequencing of miRNAs [19].

In the present study, we were able to differentiate between healthy controls and samples that were taken from Wilms tumor patients prior to chemotherapy with an accuracy of 97.5%. Several of the miRNAs that were significantly deregulated in this comparison have been associated with non-tumor diseases including miR-144* that is overexpressed in patients with active tuberculosis (TB) [20], miR-1246 that seems to play a role in the downregulation of the Down Syndrome-associated DYRK1A [21] and miR-216a that was deregulated in mouse kidney with diabetic nephropathy [22]. In addition some miRNAs that we found differentially expressed in our analysis, have previously been associated with cancer including miR-20b that is decreased in plasma vesicles of non-small cell lung cancer patients [23], miR-197 that is under-expressed in oral tumor tissue [24], and miR-144 that has been generally described as tumor specific miRNA by Wang et al. [25].

Notably, some of the miRNAs that we found differentially expressed between Wilms tumor patients and controls have previously been related to kidney cancers. Specifically miR-20a that is a member of the miR-17-92 cluster, has been correlated to alveolar rhabdomyosarcoma as reported by the Children’s Oncology Group (COG) [26] and is implicated in pathways linked to Wilms tumor [27]. There is evidence that miR-106b, which is highly expressed during nephrogenesis in nephron progenitors [28], is a predictive marker of early metastasis after nephrectomy in RCC patients [29]. The miR-17-92 cluster, also known as oncomir-1, is a miRNA polycistron that contains some of the most potent oncogenic miRNAs. The oncogenic effect of oncomir-1 was not only found in kidney cancer [30,31] but also in a broad spectrum of other malignancies including e.g. colon [32], bladder [33] or gastrointestinal cancer [34]. miR-126 a known angiogenic miRNA allowed differentiation between papillary RCC and ccRCC [35]. miR-126 has also been proposed to separate conventional from papillary renal tumors [36]. Increased levels of miR-224 have been found in ccRCCs [37].

Another interesting result of this study is that we did not find significantly deregulated miRNAs between Wilms tumor patients prior and after chemotherapy. Presently, we conclude that the tumor type seems to mainly determine the miRNA signature that is found in blood cells of the patients. Since the analyzed blood cells comprise B cells, T cells and NK cells, it is legitimate to speculate that the identified miRNA signatures reflect a tumor type specific immune response. This hypothesis awaits of course future experimental confirmation.

We are well aware that healthy adults are not the optimal control for diseased children. The improvement for blood withdrawal of healthy children is, however, a major hurdle for the time being, we do not have such an approval by the local ethic committee. However, we compared our Wilms tumor samples to samples taken from children with other tumor diseases. We have preliminary data that seems to indicate that these both groups can be separated by miRNA signatures (data not shown). These results provide further evidence for a Wilms specific miRNA signature.

## Conclusions

The present study provides first evidence for a miRNA signature found in blood cells of Wilms tumor patients. This signature shows an accuracy of 97.5% as compared to healthy controls and appears independent of chemotherapy. Since Wilms tumor patients undergo chemotherapy without prior histological analysis, an accurate blood-born biomarker like the identified miRNA signature may be helpful to avoid misdiagnosis and subsequent wrong treatment.

## Competing interests

The authors declare that they have no competing interests.

## Author’s contributions

JS planned and performed experiments, interpreted the data and wrote the manuscript; CB analyzed the data and wrote the manuscript; NN-T collected blood samples, organized the clinical data and also gave advice for planning the experiments; PL performed experiments; SD performed the qRT-PCR experiments; MB supervised the experiments; MG, NG, H-PL and AK coordinated and planned the study design; EM supervised the work and wrote the manuscript. All authors read and approved the final manuscript.

## Authors information

Andreas Keller and Eckart Meese two senior authors contributed equally to this work.

## Abbreviations

miRNA, microRNA, SIOP: Societ´e Internationale d’Oncologie P´ediatrique; COG, Children’s Oncology Group; GPOH, Gesellschaft fuer Paediatrische Haematologie und Onkologie; ccRCC, clear cell renal cell carcinoma; COPD, chronic obstructive pulmonary disease; AUC, area under the curve; ROC, receiver-operator-characteristics curve; SVM, Support Vector Machine; FDR, false discovery rate.

## Supplementary Material

Additional file 1 **Table S1.** Patient data of Wilms tumor samples. Detailed information about the diagnosis of Wilms tumor patients. Table S2. Data of control samples. Information about age and sex of control samples. Click here for file

Additional file 2 **Table S1.** Overview of significantly deregulated miRNAs in Wilms before therapy compared to normal controls. The 176 deregulated miRNAs including their fold change, p-values, and AUC values of the comparison Wims before therapy versus normal controls. Click here for file
