# Supplementary material for: Treatment-independent miRNA signature in blood of wilms tumor patients
Source: BMC Genomics. 2012 Aug 7;13:379. doi: 10.1186/1471-2164-13-379 (PMC3563587; doi:10.1186/1471-2164-13-379)
Supplement: Additional file 1 — Table S1. Patient data of Wilms tumor samples. Detailed information about the diagnosis of Wilms tumor patients. Table S2. Data of control samples. Information about age and sex of control samples. [file 1471-2164-13-379-S1.doc]

Table S1: Patient data of Wilms tumor samples.

| **Patient ID** | **Sex** | **Histology** | **Grade** | **Age [years]** | **sample taken** | **matched with** |
| --- | --- | --- | --- | --- | --- | --- |
| P109 | m | 6 | 2 | 6,22 | prior to preoperative CT |  |
| P117 | f | 7 | 2 | 5,03 | prior to preoperative CT |  |
| P120 | f | 7 | 1 | 0,80 | prior to preoperative CT | P126 |
| P125 | f | 3 | 1 | 3,53 | prior to preoperative CT |  |
| P66 | m | 8 | 3 | 5,12 | prior to preoperative CT |  |
| P68 | m | 7 | 2 | 5,82 | prior to preoperative CT |  |
| P69 | f | 5 | 1 | 9,13 | prior to preoperative CT |  |
| P88 | m | 6 | 1 | 1,85 | prior to preoperative CT | P97 |
| P90 | m | 7 | 3 | 2,52 | prior to preoperative CT | P98 |
| P92 | m | 6 | 3 | 5,00 | prior to preoperative CT | P103 |
| P21 | f | left 7 + 9, right 10 | 3 | 3,70 | prior to preoperative CT | P35 |
| P23 | f | 7 | 1 | 18,44 | prior to preoperative CT |  |
| P24 | f | 5 | 1 | 3,50 | prior to preoperative CT | P30 |
| P26 | f | 6 | 2 | 2,97 | prior to preoperative CT | P32 |
| P38 | f | 10 | 3 | 7,31 | prior to preoperative CT | P52 |
| P49 | m | 6 | 1 | 2,15 | prior to preoperative CT | P62 |
| P50 | m | 7 | 2 | 5,37 | prior to preoperative CT |  |
| P64 | m | 7 | 1 | 0,35 | prior to preoperative CT | P77 |
| P71 | f | 6 | 1 | 3,39 | prior to preoperative CT | P81 |
| P73 | m | 6 | 3 | 1,22 | prior to preoperative CT | P83 |
| P93 | f | 6 | 1 | 3,91 | prior to preoperative CT | P106 |
| P96 | m | 10 | 3 | 10,40 | prior to preoperative CT |  |
| P2 | m | 7 | 3 | 4,84 | prior to preoperative CT |  |
| P16 | m | 6 | 1 | 2,70 | after preoperative CT |  |
| P30 | f | 5 | 1 | 3,57 | after preoperative CT | P24 |
| P32 | f | 6 | 2 | 3,04 | after preoperative CT | P26 |
| P35 | f | left 7 + 9, right 10 | 3 | 3,87 | after preoperative CT | P21 |
| P52 | f | 10 | 3 | 7,42 | after preoperative CT | P38 |
| P62 | m | 6 | 1 | 2,38 | after preoperative CT | P49 |
| P77 | m | 7 | 1 | 4,92 | after preoperative CT | P64 |
| P81 | f | 6 | 1 | 0,43 | after preoperative CT | P71 |
| P83 | m | 6 | 3 | 3,46 | after preoperative CT | P73 |
| P97 | m | 6 | 1 | 1,93 | after preoperative CT | P88 |
| P98 | m | 7 | 3 | 2,63 | after preoperative CT | P90 |
| P103 | m | 6 | 3 | 5,10 | after preoperative CT | P92 |
| P105 | m | 10 | 3 | 3,99 | after preoperative CT |  |
| P106 | f | 6 | 1 | 1,30 | after preoperative CT | P93 |
| P126 | f | 7 | 1 | 0,87 | after preoperative CT | P120 |
| P56 | m | 6 | 1 | 0,35 | after preoperative CT |  |
| P74 | f | 7 | 3 | 6,41 | after preoperative CT |  |
| P75 | f | 11 | 3 | 7,87 | after preoperative CT |  |
| P58 | f | 10 | 3 | 3,70 | after preoperative CT |  |
| P94 | m | 6 | 1 | 1,06 | after preoperative CT |  |

Table S2: Data of control samples.

| **sample** | **sex** | **age** |
| --- | --- | --- |
| healthy 1* | m | 27 |
| healthy 2* | f | 41 |
| healthy 3* | f | 28 |
| healthy 4* | m | 34 |
| healthy 5* | f | 22 |
| healthy 6* | f | 58 |
| healthy 7* | m | 58 |
| healthy 8* | f | 24 |
| healthy 9* | f | 35 |
| healthy 10* | f | 23 |
| healthy 11* | f | 24 |
| healthy 12* | m | 40 |
| healthy 13* | m | 60 |
| healthy 14* | m | 57 |
| healthy 15* | f | 59 |
| healthy 16* | m | 27 |
| healthy 17* | f | 46 |
| healthy 18* | f | 27 |
| healthy 19* | f | 28 |
